# HIV/STD pattern and its associated risk factors among male STD clinic attendees in China: a foci for HIV intervention

**DOI:** 10.1186/1471-2458-11-955

**Published:** 2011-12-26

**Authors:** Qian-Qiu Wang, Xiang-Sheng Chen, Yue-Ping Yin, Guo-Jun Liang, Ning Jiang, Ting Dai, Xi-Ping Huan, Bing Yang, Qiao Liu, Yu-Jiao Zhou, Bao-Xi Wang

**Affiliations:** 1 National Center for STD Control and Prevention, China Centers for Diseases Control and Prevention, Nanjing 210042, China; 2 Jiangsu Provincial Centers for Disease Control and Prevention, Nanjing 210009, China; 3 Guangdong Provincial Center for Skin Diseases and STD Control, Guangzhou 510500, China; 4 Hainan Provincial Center for Skin Diseases and STD Control, Haikou 570206, China; 5 Guangxi Centers for Disease Control and Prevention, Nanning 542800, China

## Abstract

**Background:**

Previous studies suggested a high prevalence of STDs including HIV among female sex workers and men who have sex with men in China, but little was known about the prevalence in male patients attending public STD clinics. The aim of this study was to investigate STD patterns and HIV prevalence among male STD clinic attendees in different areas in China and the associated risk factors. The feasibility of Provider-initiated HIV testing and counseling (PITC) was evaluated as well.

**Methods:**

A cross-sectional study was conducted at 46 public STD clinics in 4 provinces in China. Between July 2009 and September 2009, a total of 3243 eligible subjects were invited to participate in an interview with a structured-questionnaire for collecting socio-demographic characteristics and sexual behavioral information. They also were asked to provide venous blood samples for serological determinations of HIV and syphilis infection, and first void urine specimens for detecting Chlamydia trachomatis and Neisseria gonorrhoeae infections,

**Results:**

Out of the 3243 eligible patients, 2951(91%) men agreed to take part in the HIV and syphilis testing. The overall prevalence rate of HIV infection was 0.7% while the rates of syphilis, *N. gonorrhoeae, C. trachomatis *infections were 10.7%, 4.3% and 6.9%, respectively, with the highest syphilis and *N. gonorrhoeae *rates in Jiangsu Province. Patients from Guangxi province, homosexual/bisexual practices and intravenous drug use were significantly associated with HIV infection in multivariate logistic regression analyses. Provider-initiated HIV testing and counseling (PITC) was well accepted by attendees, with 91% of eligible attendees agreeing to undergo HIV testing and counseling. All HIV positive patients were properly managed accordingly.

**Conclusions:**

A modest prevalence of HIV infection and substantial prevalence of other STD infections were found among male patients attending public STD clinics in China. The findings further support the introduction of HIV and syphilis PITC strategy into this important setting.

## Background

Sexually Transmitted Diseases (STDs) are a group of communicable diseases that are transmitted predominantly via sexual contact and caused by different etiologies. STDs were nearly eliminated in the 1960s in China [1]. The situation changed rapidly with the initiation of economic reform in the 1980s and STDs again became a major public health problem [2,3]. In 2005, a total of 703,001 new STD cases (including gonorrhea, syphilis, non-gonococcal urethritis, genital herpes, genital warts, chancroid and lymphogranuloma venereum) were reported. Reported syphilis cases increased sharply from 88,311 in 2004 to 358,534 in 2010. Interestingly, reported gonorrhea cases decreased significantly from 228,294 in 2004 to 105,544 in 2010 (unpublished data from National Center for STD Control, China CDC).

STD screening is useful to decrease HIV transmission because it allows identification of patients with ongoing high-risk sexual behavior and enables the treatment of STDs, which facilitate the transmission of HIV infection [4,5].

Reported epidemiological studies showed varied STD/HIV prevalence among STD clinic attendees in China, with HIV ranging from 0% to 12.6%, Chlamydia trachomatis from 6.3% to 30.3%, gonorrhea from 4.1% to 17.3% and syphilis from 9.1% to 23.2% [6-13]. None of these studies pertained to using identical laboratory methods to evaluate STDs in different areas in China. The differences in prevalence may result from varying sampling strategies and diagnostic capabilities, but also very likely reflect local endemic conditions in different geographic areas. Further understanding of STD/HIV infection prevalence in different regions in China through use of identical sampling and laboratory methods is necessary for proper planning and implementation of STD/HIV control strategies.

The first acquired immunodeficiency syndrome (AIDS) case in China was reported in June 1985 [14]. The epidemic has now entered a wide spreading phase [15,16]. Historically, the majority of reported HIV infections in China were acquired through injection drug use and use of commercial blood/plasma products. Sexually transmitted HIV cases have steadily increased and comprised more than half the reported HIV/AIDS infections since 2007 [17,18]. Heterosexual transmission of HIV accounted for 47.1% of the reported HIV cases in 2009 while homosexual transmission of HIV accounted for 8.6% of those cases. In 2009, as many as 57% of HIV-infected adults in China were estimated to be unaware of their HIV serostatus. "To little, too late" is the current HIV testing status in China. Although the Chinese government has initiated a widespread mass-screening program to enhance case finding, this program is not linked to any existing system of clinical care [19]. Client-initiated voluntary counseling and testing (VCT) has been effective in identifying substantial numbers of HIV-positive individuals. However, screening coverage remains low for many reasons including low perception of personal HIV risk, fear of stigma, and discrimination [20].

Routine opt-out provider-initiated HIV testing and counseling (PITC) services has been recommended as a newer approach to increase knowledge of HIV status. In routine opt-out PITC, HIV testing and counseling are recommended as standard components of medical care at healthcare facilities. One worldwide barrier to HIV testing has been stigma, an issue that has been increasingly acknowledged in the Chinese response to HIV. The term "opt-out" means that patients must explicitly refuse an HIV test. In this way, PITC can help to overcome stigma and to increasingly enable patients to rapidly know their HIV status. In settings where high-risk patients await health care services, such as STD clinics, failure to implement PITC is a missed opportunity for patients to benefit from counseling, prevention, early diagnosis, and referral into care and treatment for HIV infection. Currently, there are debates on whether PITC is needed among STD clinics in China. Moreover, the acceptance of PITC by both providers and patients remains unknown.

This aim of this study was to investigate STD patterns and HIV prevalence among STD clinic attendees in different areas in China and their associated risk factors. The feasibility of PITC was evaluated as well.

## Methods

### Participants

A cross-sectional study was conducted among STD clinic attendees in 46 STD clinics from 8 cities distributed in 4 provinces, including the Jiangsu, Guangdong, Hainan and Guangxi provinces of China. The 46 clinics were major medical institutions providing STD services in these cities. Nearly all of those clinics were public and located in urban areas. The outpatient loads in these clinics accounted for more than 75% of the total outpatient visits in these cities. Between July 2009 and September 2010, potential participants were identified at the STD clinics in the Guangxi, Jiangsu, and Hainan provinces. Participant identification in the Guangdong province was initiated 3 weeks later due to logistical reasons. Only those attendees who met the following criteria were included in the study: age ≥ 18 years, male gender, and either: presentation with STD related complaints, history of high-risk sexual behaviors (such as exchange sexual activity or multiple sexual partners), or history of one or more sexual partners with a STD. Patients seeing physicians for sexual dysfunction or for skin diseases unrelated to STDs were excluded. To ensure privacy, the interviews were conducted in a separate space within the clinic. Interviewers were trained in techniques for conducting research in the sensitive topics of sexual health. Regular meetings were held to discuss the progress of the survey as well as any special problems encountered. The purpose and procedures of the study were explained to each study participant Participants who were willing and able to complete the questionnaire by themselves were given written questionnaires to complete themselves. In such cases, interviewers were available to help with answering participants' questions if needed.

After completing the questionnaire, the participants were provided with information about HIV and other STD testing, and subsequently were asked to undergo HIV and STD testing. Participants were informed of the syphilis test results and the HIV test results (if negative) by the next day. Results of confirmatory HIV tests, Neisseria gonorrhoeae tests, and Chlamydia trachomatis tests were provided 10 days later. If necessary, treatment of individual STDs was initiated on the same day that results were disclosed. After-HIV-testing, counseling was provided to participants according to their sero-status.

### Study measures

Structured questionnaire-based interviews provided socio-demographic characteristics, sexual health services accepted over the past year, and sexual behavior information. The questionnaires were confidential, but not anonymous, and subsequent diagnostic data was linked to the behavioral questionnaire.

Socio-demographic variables of interest included age, ethnicity, educational level, marital status, residency, and living arrangement status. Sexual behavioral information mainly included sexual orientation, frequency of condom use with regular sex partner, whether condoms were used with regular sex partner(s) in the last sexual encounter, presence of recent encounters with casual sexual partner(s), and/or with FSW (and condom usage accordingly). Drug use status was also evaluated.

### Laboratory testing

After completing the questionnaire, the participants were provided with information about HIV and other STD testing, and were requested to undergo testing for syphilis, HIV, *Chlamydia trachomatis*, and *Neisseria gonorrhoeae*. Blood was drawn for syphilis testing in local hospitals. The study team provided identical testing reagents to local hospitals. The toluidine red unheated serum test (TRUST, Rongsheng Biotech Inc, Shanghai, China) was used for syphilis screening and quantitative analysis. *Treponema pallidum *particle agglutination test (TPPA; Fujirebio Inc., Tokyo, Japan) or *Treponema pallidum *-enzyme-linked immunoassay (TP-ELISA, Wansheng Biotech Inc, Beijing, China) was used for syphilis test confirmation. HIV, *Chlamydia trachomatis*, and *Neisseria gonorrhoeae *testing were performed in the National Center for STD Control, China CDC. For HIV, a screening test algorithm using ELISA (Wansheng Biotech Inc, Beijing, China) and a confirmatory test using the Western blot assay (HIV Blot 2.2, Genelabs Diagnostics, Singapore) were used. First void urine specimens were tested for *Chlamydia trachomatis *and *Neisseria gonorrhoeae *by means of polymerase chain reaction (PCR) according to the manufacturer's instructions (Amplicor Chlamydia trachomatis/Neisseria gonorrhoeae Test, Roche Diagnostic Corp, USA).

This study was approved by the Ethics Committee of the National Center for STD Control, China CDC.

### Statistical analysis

Data were recorded using Microsoft Excel™ (Edition 2003, Microsoft Corporation, Redmond, WA, USA) and validated through double entry. Univariate and multivariable logistic regression analyses were performed using Statistical Package for the Social Sciences (SPSS15.01™, SPSS Inc., Chicago, IL, USA) to test for evidence of an association between the categorical variables. Odds ratios (ORs) and their corresponding 95% confidence intervals (CI) were produced to present the risk factors for different behaviors. Multiple regression analyses were performed to adjust ORs for potential confounders. Only variables that were significant in univariate analyses at *P *< 0.1 were included in multivariable logistic regression models for selecting the independent risk factors.

## Results

### Proportion of testing and STD/HIV prevalence

During the period from July 2009 and September 2009, 2951(91.0%) of 3243 eligible attendees agreed to undergo HIV and syphilis testing, among which 2041(69.2%) attendees also provided first void urine for *C. trachomatis *and *N. gonorrhoeae *testing. Prevalence of syphilis, *N. gonorrhoeae, C. trachomatis*, and HIV was 10.7%, 4.3%, 6.9% and 0.7% respectively. Among the 21 HIV-positive patients, 19%(4/21) were syphilis sero-positive, 7.1% (1/14) were N. gonorrhoeae positive and 14.3% (2/14) were C. trachomatis positive. STDs and HIV prevalence rate by different areas are shown in Table 1. Syphilis and *N. gonorrhoeae *rates were highest in Jiangsu Province.

**Table 1:** STD/HIV infection by Provinces

	Overall	Jiangsu	Guangxi	Guangdong	Hainan
**Syphilis**	10.7 (315/2951)	19.4 (202/1041)	6 (47/788)	8.9 (35/394)	4.3 (31/729)

**N.gonorrhoea**	4.3 (88/2041)	8 (58/725)	2.1(11/533)	4.1 (15/364)	1 (4/419)

**C. trachomatis**	6.9 (140/2041)	5.9 (43/725)	6.9 (37/533)	8.2 (30/364)	7.2(30/419)

**HIV**	0.7 (21/2951)	0.6(6/1041)	1.8(14/788)	0(0/394)	0.1 (1/729)

### Study population and sexual behavioral factors

Demographic characteristics of the study population were shown in Table 2. The age of study patients ranged from 18 to 88 years and the median age was 34.8 years. The most-common ethnic group was Han (96.5%), and 68.6% of participants were married. The majority was composed of participants with education levels equal to or less than middle school (56.0%). A total of 77.5% of participants resided locally to the investigating city.

**Table 2:** Demographic Characteristics of Population Tested (N = 2951)

Characteristics	% (No.) or Mean With Range
**Median age (range), in years**	34.8 (18-88)

**Ethnicity**	

Han	96.5 (2828/2932)

Others	3.5 (104/2932)

**Education Level**	

Primary school equivalent or less	11.5 (338/2944)

Middle school	44.5(1309/2944)

High school	28.6(843/2944)

junior college	15.4(454/2944)

**Marital status**	

Single	28.4 (835/2937)

Married	68.7 (2014/2937)

Divorced/Widowed	3.0 (88/2937)

**Living Arrangement Status**	

Living alone	15.1 (445/2939)

Living with spouse	60.9 (1789/2939)

Cohabited with other female	3.2 (93/2939)

Living with male	5.5 (163/2939)

Living with family/others	15.2 (449/2939)

**Residency**	

Local city	77.5 (2247/2899)

Local province(not local city)	9.0 (260/2899)

Other province	13.5 (392/2899)

It was shown that 45.5% of patients reported never using condoms, 48.9% sometimes used condoms, and only 5.5% consistently used condom with their steady sex partners within the last year. 24.8% of participants used condoms at their last sexual encounter with their steady sex partners. 46.0% of participants acknowledged that they had visited female sex workers (FSWs) within the last 3 months. The proportion of condom use with FSWs at last sexual encounter was 31.8%. At same time, up to 25.7% acknowledged having a non-FSW casual sex partner(s) within the last 3 months, of which only 30.1% used condoms at their last sexual encounter. Twenty-six (0.9%) patients were homosexual/bisexual (Table 3). 3.9% (116/2941) of participants reported drug abuse in the last year. Fifteen patients reported injection drug use (IDU) in last 6 months, of whom 3 patients reported sharing needles for drug injection.

**Table 3:** Sexual Behavior of the Male STD Clinic Patients

Characteristics	% (No.) or Indicated Value
**Condom use frequency with regular sex partner**	

Never	45.5 (1100/2416)

Sometimes	48.9 (1182/2416)

Always	5.5 (134/2416)

**Condom use with regular sex partner last time**	

Yes	24.8 (579/2412)

No	75.2 (1815/2412)

**Whether visiting FSW in the last three months**	

Yes	46.2 (1320/2886)

No	53.8 (1556/2886)

**Condom usage with FSW last time**	

Yes	31.8 (419/1319)

No	68.2 (900/1319)

**Whether had casual sexual partner in the last three months**	

Yes	25.7 (736/2865)

No	74.3 (2129/2865)

**Condom usage with casual sexual partner last time**	

Yes	30.1 (228/757)

No	69.9 (529/757)

**Homosexual/bisexual**	

Yes	0.9 (26/2865)

No	97.3 (2839/2865)

**IDU in the last 6 months**	

Yes	0.5 (15/2902)

No	99.5 (2887/2902)

### The feasibility of providers-initiated HIV testing

A total of 3243 eligible attendees were screened in clinics. With informed consent, 2951(91.0%) patients agreed to provide blood for HIV and syphilis testing and were included in the final analysis. Twenty-one (0.7%) were confirmed to be HIV positive and were managed either in the investigating clinics with help from the local CDC or were referred to specialized hospitals for HIV management.

### HIV/STD associated risk factors

An extremely low proportion (6.2%, 182/2933) of the participants had ever received HIV testing and counseling. Of the 21 HIV positive patients, 66.7% (14) reported no STD-related symptoms in the last year. Most of the HIV positive patients were from the Guangxi and Jiangsu Provinces. Fourteen (66.7%) of the HIV positive patients were married and 12 lived with their spouses, none used condoms consistently. Six patients had visited FSWs in the past 3 months and none had used condoms in their last sex encounters. One HIV positive patient had casual sex with a woman other than a FSW in the past 3 months.

In univariate logistic regression analyses, the following participant characteristics were significantly associated with HIV infection: being from the Guangxi province, age ≥ 40 years, lower education level (elementary school or illiterate), being homosexual/bisexual, and IDU. Multivariate logistic regression analyses showed that factors strongly associated with HIV infection were: investigation site (patients from Guangxi Province, AOR, 12.3, 95% CI, 1.5-97.4), being homosexual/bisexual (AOR, 16.9, 95% CI, 3.2-90.6) and IDU (AOR, 19.1, 95% CI, 2.1-176.1) (Table 4).

**Table 4:** Risk factors associated with HIV/STD among male STD clinic attendees

	HIV		STD	
	
Variable	Univariate OR(95%CI)	Multivariate AOR(95%CI)	Univariate OR(95%CI)	Multivariate AOR(95%CI)
**Original site**				

JiangSu Province	4.2 (0.51-35.1)	2.8 (0.3-25.3)	3.9(2.9-5.2)**	3.8(2.8-5.3) **

Guangxi Province	13.2 (1.7-100.3)*	12.3(1.5-97.4)*	1.4(1.0-2.0)*	1.4(1.0-2.1) *

GuangDong Province	0	0	2.5(1.7-3.6)**	2.7(1.7-3.6)**

HaiNan Province	1	1	1	1

**Age (Years)**				

18-25	1		1	1

26-45	2.1(0.46-9.4)		1.6(1.2-2.0)**	1.6(1.1-2.3)**

> 45	5.7(1.2-27.0) *		2.3(1.7-3.1)**	2.0(1.3-3.1)**

**Ethnicity:**				

Han			1	

Others			0.3(0.2-0.7) **	

**Education Level**				

Primary school equivalent or less	11.0(1.4-88.2) *		1.4(0.9-2.0)	1.2(0.8-2.0)

Middle school	2.8(0.3-22.3)		1.5(1.1-2.0)	1.6(1.2-2.3) **

High school	2.2(0.2-19.5)		1.5(1.1-2.0)	1.5(1.1-2.2) *

College or higher	1		1	1

**Marital status**				

Single			1	

Married			1.4(1.1-1.7)*	

Divorced/widowed			1.1(0.6-2.0)	

**Visiting FSW in the last 3 month and no condom use last time**				

Yes	0.9(0.3-2.3)		1.3(1.1-1.6) **	1.4(1.1-1.7) **

No	1		1	1

**Have casual sex partnet other than FSW in the last 3 month and no condom use last time**				

Yes			1.2(1.0-1.6) *	1.3(1.0-1.7) *

No			1	1

**Homosexual/bisexual**				

Yes	13.1(2.9-59.4) **	16.9 (3.2-90.6) **		

No	1	1		1

**IDU in the last 6 months**				

Yes	10.2 (1.3-81.6) *	19.1(2.1-176.1)*		

No	1	1		


CI = confidence interval; OR = odds ratio; AOR = adjusted odds ratio

* *P*< 0.05 ** *P *< 0.01

For the combination of the 3 STDs (syphilis, *C. trachomatis *and *N. gonorrhoeae*), our multivariable logistic regression suggested that investigation site, older age, lower education level, unsafe sex with a FSW (defined as visiting a FSW in the past 3 months and no condom use in their last sexual encounter), and unsafe sex with a non-FSW casual sex partner (defined as having a non-FSW casual sex partner in the past 3 months and no condom use in their last sexual encounter) were independent risks factors (Table 4).

## Discussion

This large-scale cross-sectional study using identical laboratory methods among different areas in China demonstrates a high prevalence of STDs in patients attending STD clinics. Overall prevalence rates of syphilis, *N. gonorrhoeae, C. trachomatis *and HIV were 10.7%, 4.3%, 6.9% and 0.7% respectively. After decades of exceedingly low STD rates in China, STDs had become widely-spread through commercial sex work and bridging populations. Syphilis prevalence is much higher than *N. gonorrhoeae *prevalence. This result is consistent with national surveillance data on STD infection. During the past decades, the incidence rate of gonorrhea had declined while the syphilis incidence rate increased dramatically [2,21,22]. This might be attributed to the relative ease of detection and treatment of gonorrhea. Meanwhile, under-reporting of gonorrhea cases by health care providers may have occurred due to the common practices of self-treatment or visiting pharmacies for treatment of suspected gonorrhea in people with high risk sexual behaviors. Syphilis prevalence (10.7%) in China greatly exceeds most other reports in developed countries and India [23-25]. However, the prevalence is comparable to recent studies among STD clinic attendees in the Guangxi province (11.9%), and the Guizhou and Anhui (11.6%) provinces in China [7,26]. Additionally, in our study, syphilis prevalence was not distributed equally across these provinces. The highest prevalence of syphilis was found in Jiangsu (19.4%), followed by Guangdong (8.8%), Guangxi (6.0%), and Hainan (4.3%) provinces. The highest gonorrhea infection rate was also found in Jiangsu province. This difference is probably due to sociocultural and economic determinants. Jiangsu had a GDP per capita of $6,475 USD in 2010, exceeded the other 3 investigating provinces. It has been shown that the highest incidences of STDs are in China's wealthiest areas [27]. *C. trachomatis *prevalence has been studied in several areas of the world, with the prevalence rate among STD clinics varying from 4.6% in Jordan to 13.1% in Brazil [28,29]. With a much higher syphilis positive rate than *C. trachomatis *rate in our study, the STD pattern in China is different from others areas [30]. These results strongly support syphilis screening for every STD clinic attendee, which is highlighted in China's Plan for Syphilis Prevention and Control (2010-2020).

The high STD prevalence alongside low condom use rates among these clinic attendees emphasizes the gravity of China's STD/HIV epidemic. Only a few participants (2.2%, 65/2933) had ever been offered partner notification services. Partner notification and treatment are important parts of a STD control strategy as one of the few available means of reaching individuals with asymptomatic STDs. Partner notification is also an opportunity for health education and promotion of condom use, and should be initiated and strengthened in STD clinics [31].

Overall, 21(0.7%) patients were found to be HIV positive. There was considerable geographical variation in HIV prevalence, ranging from 0% in Guangdong and 0.1% in Hainan to 0.6% in Jiangsu and 1.8% in Guangxi. According to the AIDS Prevalence Report of the China Ministry of Health (MOH), five high HIV prevalence provinces accounted for 80.5% of the total HIV/AIDS cases in the country [32]. Guangxi had a total of 20,604 reported HIV infections, ranking third highest among the provinces. In another study in Guangxi, the HIV prevalence was similar (1.2%) to that of our study [33]. No HIV patients were found in Guangdong despite a moderate HIV epidemic in Guangdong, perhaps due to insufficient patients recruitment in this province. No significant association was shown between HIV and sexual risk behaviors, perhaps due to a small HIV positive sample size.

In our study, we found that investigation site, older age, lower education level, and unsafe sexual behavior with casual partners were factors that were independently associated with a STD. Notably, older participants had a higher risk for STD, which is different from previous reports which often showed a higher STD prevalence in younger participants. Due to limited economic capabilities, older men may tend to visit low-tier FSWs, where STD prevalence is substantially higher than in other tiers,. Our previous study showed a syphilis positive rate of more than 30% in low-tier FSWs, which is much higher than that in middle or high-tier FSWs. Other recent studies in China raised attention due to significant increases in the number of older patients with syphilis or HIV since 2005.

Given the rising prevalence rates of STDs, China faces a rapidly increasing risk of HIV infection through sexual transmission (heterosexual and homosexual) from unprotected sexual behavior. 323,252 HIV cases were reported in China as of the end of 2009. Furthermore, the number of HIV-positive people indicated by the existing registration system may be only a small proportion of the total number of HIV-infected people. Therefore, identifying the hidden population of HIV-infected people is critical for successful HIV control. Despite efforts to screen high-risk populations for HIV through local CDC outreach programs and voluntary counseling and testing clinics, HIV-positive individuals are still diagnosed substantially later in their disease course than individuals in North America. Thus, urgent expansion of the WHO- recommended PITC strategy in high-risk cohorts and in high HIV-prevalence locations should be considered. This strategy is cost-effective. As HIV programs are scaled up and technical capacity grows in China, local public STD clinics provide an excellent location for targeted HIV testing and prevention. STD clinic attendees represent a major risk group for HIV acquisition and transmission and can easily be approached. Patients at STD clinics may be willing to undergo HIV testing in light of their high risk sexual behaviors and may desire to be tested in the convenient and confidential clinic settings. In this study, PITC was well accepted among male STD clinic attendees. The percentage of individuals who agreed to HIV testing was comparable to that reported in another study among tuberculosis patients in China [34]. This pilot study demonstrates that it is feasible to apply PITC-based HIV testing in STD clinics. Currently there are debates on whether PITC is needed. Sixty-seven percent (14/21) of the HIV positive patients reported no STD-related symptoms in last year. If they had not participated in our study, most of them would have remained undiagnosed. PITC could lead to many benefits: increased patient awareness of HIV status, adequate counseling with education on HIV prevention, and earlier access to care with earlier initiation of antiretroviral treatment. STD clinics provide an excellent venue for HIV intervention. At same time, health-care providers need to consider ethical issues. They should inform patients that HIV testing is a routine clinic practice to decrease stigma, but should avoid compulsory testing. They also need to keep patients' information confidential. In addition, HIV testing must be accompanied by HIV treatment, care, and support, and cannot be implemented in isolation.

The limitations of the study are that bias may exist (such as participation, reporting, and recall bias), just as in other cross-sectional studies. The participants of the study attended public STD clinics during a specific period of time. The 46 clinics represented most (more than 75%) of the total outpatient visit in these cities. It should be noted that only 60.6%-68.3% male patients visited public STD clinics when they suspected STD infection [35,36]. Therefore, caution should be taken before generalizing the findings of the study to the entire population in China.

## Conclusions

There is a high prevalence of STD among male STD clients in China. The prevalence of specific infections varied among areas. HIV was found in three of the four investigation provinces. We strongly recommend that PITC should be started in STD clinics because they are good venues for HIV intervention.

## Competing interests

The authors declare that they have no competing interests.

## Authors' contributions

QQW had full access to all of the data in the study and takes responsibility for the integrity of the data. QQW, XSC, BXW,GJL participated in study concept and design, interpreted results and supervision. YYP provided laboratory support for the project. NJ is the coordinator of the project. QQW and DT were responsible for data analysis and manuscript drafting. XPH, BY, QL, and YJZ supervised the study procedures on site and provided data. All the authors read and approved the final manuscript.

## Pre-publication history

The pre-publication history for this paper can be accessed here:

http://www.biomedcentral.com/1471-2458/11/955/prepub
